# Diagnostic performance of attenuated total reflection Fourier-transform infrared spectroscopy for detecting COVID-19 from routine nasopharyngeal swab samples

**DOI:** 10.1038/s41598-022-24751-z

**Published:** 2022-11-27

**Authors:** Helinä Heino, Lassi Rieppo, Tuija Männistö, Mikko J. Sillanpää, Vesa Mäntynen, Simo Saarakkala

**Affiliations:** 1 Research Unit of Medical Imaging, Physics and Technology, University of Oulu, Oulu, Finland; 2 Northern Finland Laboratory Centre NordLab, Oulu, Finland; 3 Research Unit of Mathematical Sciences, University of Oulu, Oulu, Finland; 4 Department of Diagnostic Radiology, Oulu University Hospital, Oulu, Finland

**Keywords:** Viral infection, Infrared spectroscopy

## Abstract

Attenuated total reflection Fourier-transform infrared (ATR-FTIR) spectroscopy coupled with machine learning-based partial least squares discriminant analysis (PLS-DA) was applied to study if severe acute respiratory syndrome coronavirus 2 (SARS-CoV-2) could be detected from nasopharyngeal swab samples originally collected for polymerase chain reaction (PCR) analysis. Our retrospective study included 558 positive and 558 negative samples collected from Northern Finland. Overall, we found moderate diagnostic performance for ATR-FTIR when PCR analysis was used as the gold standard: the average area under the receiver operating characteristics curve (AUROC) was 0.67–0.68 (min. 0.65, max. 0.69) with 20, 10 and 5 k-fold cross validations. Mean accuracy, sensitivity and specificity was 0.62–0.63 (min. 0.60, max. 0.65), 0.61 (min. 0.58, max. 0.65) and 0.64 (min. 0.59, max. 0.67) with 20, 10 and 5 k-fold cross validations. As a conclusion, our study with relatively large sample set clearly indicate that measured ATR-FTIR spectrum contains specific information for SARS-CoV-2 infection (P < 0.001 for AUROC in label permutation test). However, the diagnostic performance of ATR-FTIR remained only moderate, potentially due to low concentration of viral particles in the transport medium. Further studies are needed before ATR-FTIR can be recommended for fast screening of SARS-CoV-2 from nasopharyngeal swab samples.

## Introduction

The Global COVID-19 pandemic has raised a desperate need for an accurate, fast and cheap test to efficiently detect infected people to prevent the spreading of coronavirus^1,2^. Polymerase chain reaction (PCR) method is the gold standard for detecting severe acute respiratory syndrome coronavirus 2 (SARS-CoV-2) from respiratory secretions^3^. However, PCR is not the best modality for quick screening, as it requires certain sample preparation and transportation to centralized laboratories. Therefore, new cost-effective tests for SARS-CoV-2 are being developed. Furthermore, in the future, the possibility to adjust cost-effective test to the novel viruses could provide a way to avoid new infectious diseases developing to a pandemic state.

According to the scientific literature, attenuated total reflection Fourier-transform infrared (ATR-FTIR) spectroscopy is a potentially suitable method for the fast detection of SARS-CoV-2 infection^4–6^. In ATR-FTIR measurement, infrared (IR) light is guided to a sample to measure how the sample molecules interact with the IR light. Collected data shows molecular bond vibrations related to the sample chemical composition, i.e., revealing the chemical fingerprint for the studied sample. Besides fast measurement time in ATR-FTIR method (a few minutes), several ATR-FTIR equipments are already portable, which could allow analysis of the biological samples directly in the public places like border control stations, shopping centres and airports.

ATR-FTIR has been used earlier in diverse studies to detect different conditions from human liquid biopsies, such as breast cancer from saliva^7^, dengue fever from blood and serum^8^ and hepatitis B and C from sera^9^. During the last year, ATR-FTIR method has already been applied to detect SARS-CoV-2 from blood (both in serum and plasma^10,11^) and saliva samples^12,13^. Even though results from these studies are extremely promising, the number of investigated samples is typically relatively small. For example, in the study by Barauna et al. excellent results were obtained for detecting SARS-CoV-2 infection directly from the pharyngeal swab samples (accuracy, sensitivity, and specificity of 90%, 95%, and 89%, respectively) within a total of 181 samples^12^. Furthermore, in the study by Nogueira et al., authors had 65 nasopharyngeal swab samples in viral transport medium 1 (sensitivity 84%, specificity 66% and accuracy 76.9%) and 178 nasopharyngeal swab samples in viral transport medium 2 (sensitivity 87%, specificity 64% and accuracy 78.4%)^14^. Despite these promising preliminary results, more research is needed, especially with larger sample size, in order to judge the true diagnostic performance of the ATR-FTIR method in realistic clinical scenario.

Our aim was to investigate the diagnostic performance of the ATR-FTIR spectroscopy to detect the SARS-CoV-2 infection from the same routine nasopharyngeal swab samples that are used in PCR. Compared to earlier studies, our analyzed sample set contained 558 positive and 558 negative samples, making it the largest ATR-FTIR study with balanced dataset so far to detect the SARS-CoV-2 from the very same nasopharyngeal swab samples that were originally collected for the PCR.

## Materials and methods

### Ethical permissions to study nasopharyngeal swab samples

Nasopharyngeal swab samples were originally collected by Finnish public healthcare in the Northern Ostrobothnia region, Northern Finland, for conducting PCR tests to detect patients with SARS-CoV-2 infection. Thus, the collection of nasopharyngeal swab samples was not part of our study, instead our study was retrospective trying to detect the SARS-CoV-2 infection from the routine nasopharyngeal swab samples by using ATR-FTIR spectroscopy. As our study being retrospective, it was not possible to obtain detailed patient information like diabetes mellitus, status of other viral infections, or other medical records.

The ethical permissions were obtained both locally from the Ethical Committee of North Ostrobothnia’s Hospital District (meeting agenda number and section sign: EETMK: 95/2020 (216 §)) as well as nationally from the Finnish Medicines Agency (www.fimea.fi) (permission identifier: FIMEA/2021/003461). A total of 558 negative and 558 positive samples were included in this study from January 1st 2020 to November 24th 2020. As this study used pre-existing diagnostic specimens, the requirement for informed consent was waived by the Finnish Medicine Agency under the provisions outlined in Sect. 21a of the Tissues Act (2012). Also, we confirm that all research was performed in accordance with relevant guidelines and regulations.

### Nasopharyngeal swab samples and ATR-FTIR measurement

The studied nasopharyngeal swab samples were originally collected by Finnish public healthcare for conducting PCR tests to detect patients with SARS-CoV-2 infection. Residues from these nasopharyngeal swab samples were stored in a freezer (− 20 degrees) and thawed prior to the ATR-FTIR measurements. A total of 558 negative and 558 positive samples were measured in this study.

**Figure 1:**
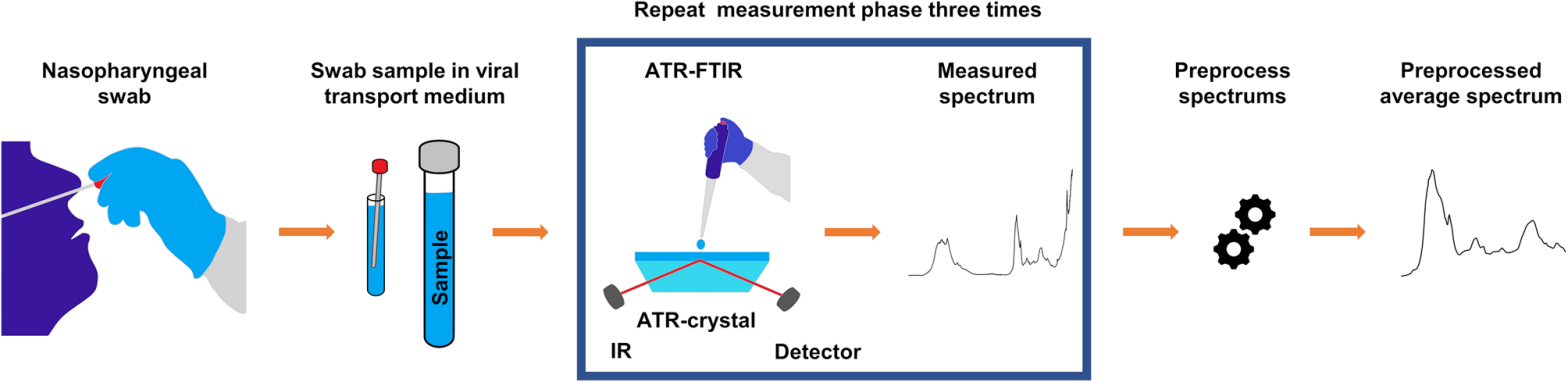
Data acquisition. A routine nasopharyngeal swab sample was dissolved into the viral transport medium, analyzed with PCR, and the remnant from that sample was stored in the freezer. The frozen swab sample was thawed and inactivated by adding a viral lysis buffer liquid before the ATR-FTIR measurements. The measurement was conducted by pipetting a one drop from sample solution containing the nasopharyngeal swab sample, the viral lysis buffer and the viral transport medium onto the ATR crystal. Pipetting and measurement was repeated three times to obtain three spectra from each sample. Those three measured spectra were finally averaged so that there was one representative spectrum from each nasopharyngeal swab sample. Before ATR-FTIR measurement, the sample was vortexed to have as homogeneous material distribution as possible, and spun with centrifuge to set the whole sample to the bottom of the tube.

The PCR analysis, used as the gold standard for the ATR-FTIR measurements, was conducted by the Northern Finland Laboratory Centre NordLab (www.nordlab.fi), Oulu, Finland. The nasopharyngeal swab samples were collected with a flocked swab and immediately immersed in viral transport medium. During the sample collection period patients were instructed to be tested for infection with SARS-CoV-2, if they had any symptoms indicative of COVID-19. Asymptomatic individuals were tested in Finland upon discretion of physicians working in disease control. The samples were then transported in room temperature to the laboratory performing the analyses. The laboratory had no information on patients’ symptoms. The PCR methods were performed according to instructions. Before the PCR measurements, the swab samples were dissolved into the viral transport medium, and the residues from these samples were stored in the freezer for ATR-FTIR measurements. Before ATR-FTIR all the swab samples were inactivated with viral lysis buffer liquid. Bruker Alpha II FTIR spectrometer (Bruker Optics GmbH, Ettlingen, Germany) equipped with an ATR module (Platinum ATR, Bruker Optics GmbH, Ettlingen, Germany) was used to perform ATR-FTIR measurement one-by-one for the every sample after thawing. The spectral resolution was set to 2 cm^−1^, the number of scans to 64, and the collected spectral range was 4000 to 400 cm^−1^. A droplet (volume: 1 µl) of the sample solution was pipetted onto the ATR crystal (Fig. 1). A repeat measurement was started immediately after the sample droplet was placed onto the ATR crystal to collect a total of 10 successive spectra. The spectrum from the last measurement was selected into the final data analysis to ensure that the water has evaporated. This measurement procedure was repeated three times for each sample to minimize differences due to pipetting process. The ATR-crystal was cleaned with ethanol and Virkon disinfectant after every measurement procedure when the measurement was completed to prevent sample contamination.

### Spectral preprocessing

The region of 1800–900 cm^−1^ is known as fingerprint region in IR-spectrums observed from biological samples, as it reveals important information related to structures in biological specimens^4^. The fingerprint region contains spectral peaks connected to lipid, Amide I, Amide II, Amide III, Carbohydrate, asymmetric phosphate (PO_2_^−^) stretching vibrations, symmetric phosphate (PO_2_^−^) stretching vibrations, glycogen and protein phosphorylation^15^. Obviously, differences in the fingerprint region are also expected to make difference between COVID-19 negative and COVID-19 positive cases. Thus, the ATR-FTIR spectra were truncated to the fingerprint region of 1800–900 cm^−1^, vector normalized, and the three spectra of each sample were averaged to create one average spectrum per sample. In addition, the spectral region of 1490–1180 cm^−1^ was also analyzed in the same way as fingerprint spectrum region, as preliminary testing indicated similar performance in classification, as with fingerprint spectral region. The spectral region of 1490–1180 cm^−1^ was found from preliminary analysis where manual testing was performed to study if there is smaller area inside the fingerprint region which could contain enough information to classify spectrums. Performance of the classifying model was followed to determine if studied smaller region was enough informative. Preprocessing steps were performed with Anaconda3 (Conda 4.8.3 package manager and Python version 3.8.3) using NumPy, opusFC and scikit-learn packages^16^.

### PLS-DA and data analysis

Partial least squares discriminant analysis (PLS-DA) is a popular method for classification for multivariate datasets. PLS-DA performs dimensionality reduction by creating latent variables, i.e. PLS components, by maximizing the covariance between the new predictor and response scores^17–19^. According to authors’ own experience and literature^9,10^ PLS-DA is particularly suitable for classification of FTIR spectra, and it was therefore selected as the primary data analysis approach in this study.

Anaconda3 (Conda 4.8.3 package manager and Python version 3.8.3) and PLS Regression package of the scikit-learn library was used to create the PLS-DA model^16^. The performance of the model was studied by using cross-validation with k-fold values 5, 10 and 20. Every k-fold cross-validation was repeated 100 times with different random seed initializations to make sure that the results are not affected by the random seed choice used to divide data into training and validation sets. The presented results have been calculated as average, minimum, and maximum values of the repeated k-fold cross validations. Receiver operating characteristic (ROC) curve, area under the receiver operating characteristics (AUROC) curve, accuracy, sensitivity, specificity, precision, and confusion matrix were used to assess the model performance^20,21^.

We repeated the same predictive analysis 1000 times by randomly permuting sample labels in each k-fold case. This permutation analysis was performed to obtain an empirical null distribution of the AUROC values, where we can then see how the extremal position (quantile), the corresponding AUROC value, obtained with the real data, can find in this distribution. This was done to show that the PLS-DA model have the real skill to differentiate between the positive and negative sample groups, and thus it is not possible to obtain a similar magnitude of AUROC values by chance by randomly dividing samples into the negative and positive classes. P-values were calculated by using test 1 according to Ojala and Garriga^22^. For permutation tests, see also Efron and Hastie^23^. In practice, one random seed used to divide data into the training and validation sets was chosen, and the 1000 permutations were performed to achieve the null distribution of AUROC-values for each k-fold training in question, meaning k-fold 20, 10 or 5^23^.

## Results

**Figure 2:**
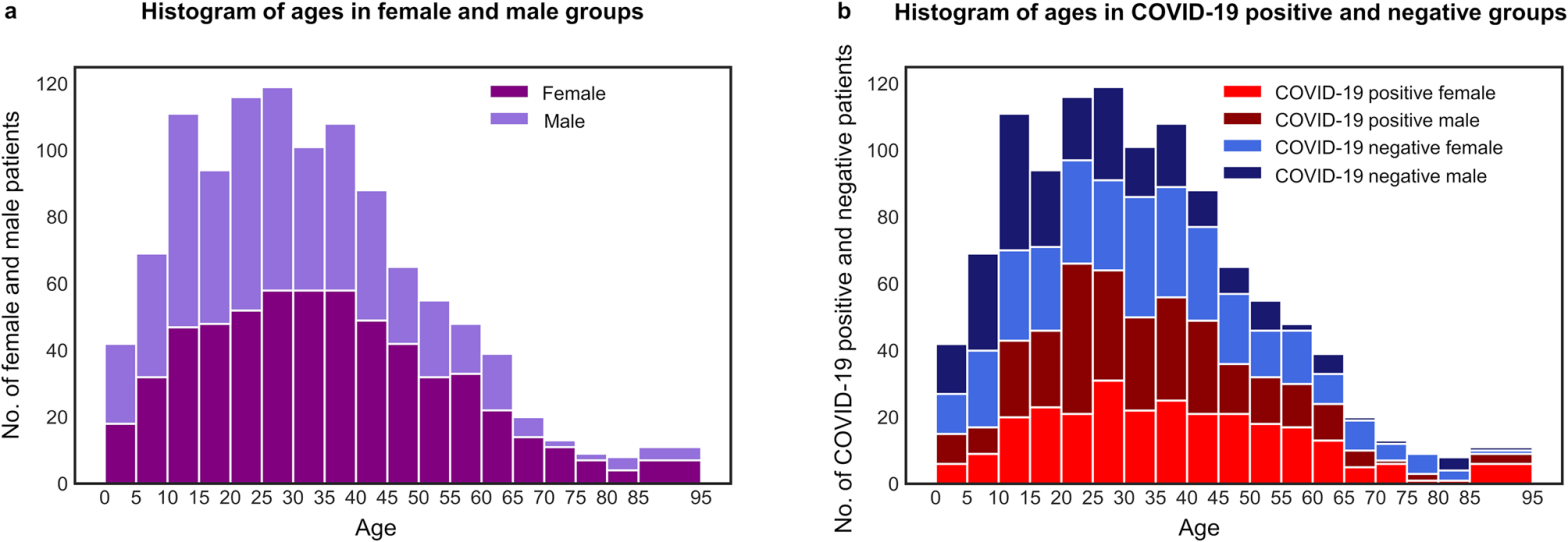
Patient histograms. The analyzed dataset contained 1116 separate nasopharyngeal swab samples from 592 female (of whom 266 COVID-19 positive and 326 COVID-19 negative) and 524 male (of whom 292 COVID-19 positive and 232 COVID-19 negative) patients with ages between 0 and 94 years (mean ± standard deviation, 32 years ± 19 years). The terms female and male refers to the biological sex. (**a**) Histogram describing distribution of female and male patients with different ages in analyzed dataset. (**b**) Histogram describing COVID-19 disease distribution of patients in female and male groups with different ages.

**Figure 3:**
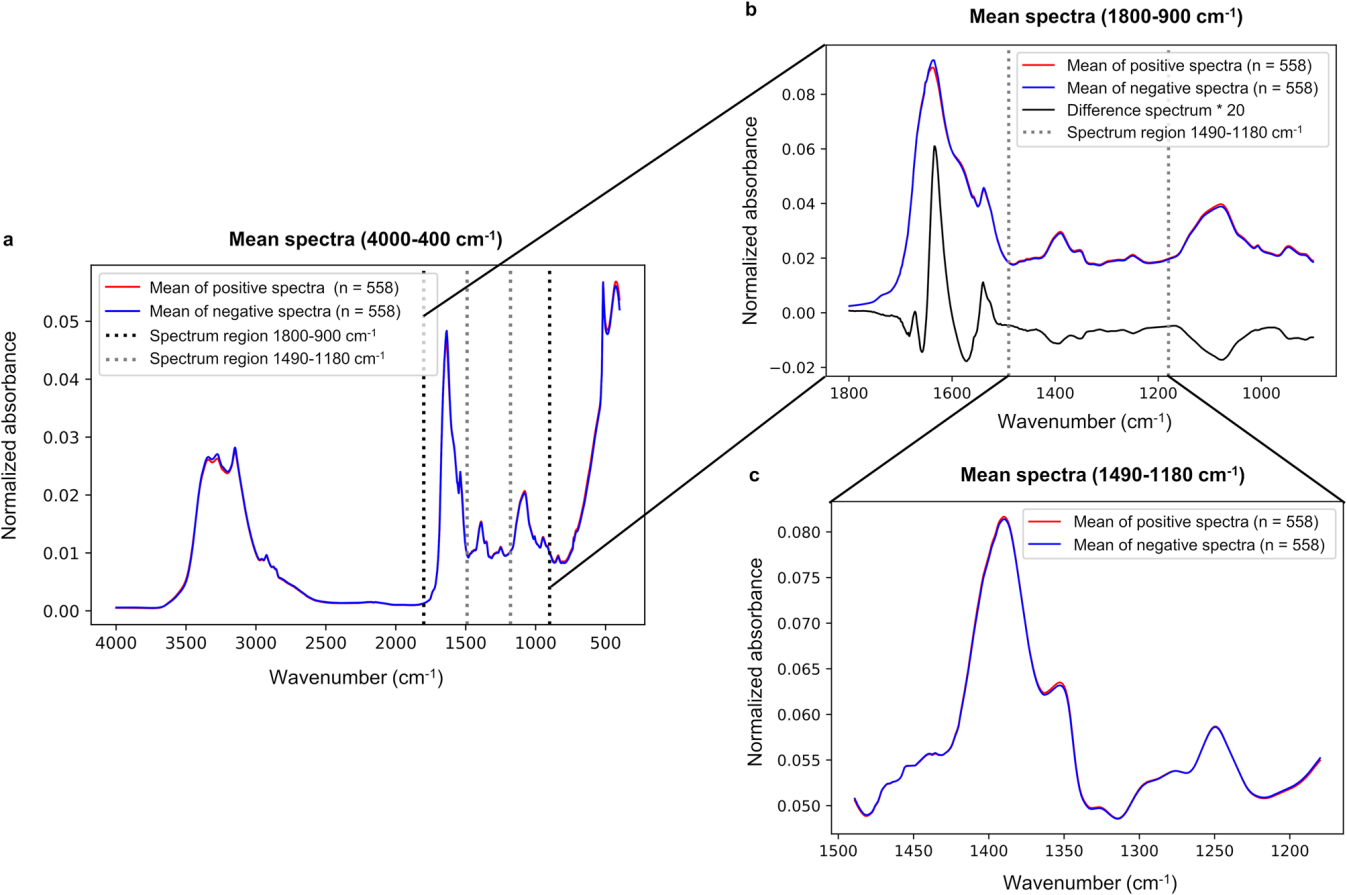
Mean spectra of positive and negative groups from different spectral regions. Vector normalization was applied before calculating the mean spectra. (**a**) Full mean spectra (4000–400 cm^−1^) from positive and negative groups. Also, spectrum regions of 1800–900 cm^−1^ and 1490–1180 cm^−1^ are marked. (**b**) Mean spectra from positive and negative groups from the fingerprint region, or spectrum region of 1800–900 cm^−1^. Difference spectrum from the fingerprint region multiplied by factor of 20 is presented to illustrate differences between the sample groups in the fingerprint region. Spectral region of 1490–1180 cm^−1^ is also marked, (**c**) Mean spectra from positive and negative groups from the spectrum region of 1490–1180 cm^−1^. Only minor spectral changes were visually observed between the sample groups in the spectral region of 1490–1180 cm^−1^, although the diagnostic performance in the cross-validation setup was almost identical than with the whole fingerprint region (Tables 1, 2 for the spectral region of 1490–1180 cm^−1^).

**Figure 4:**
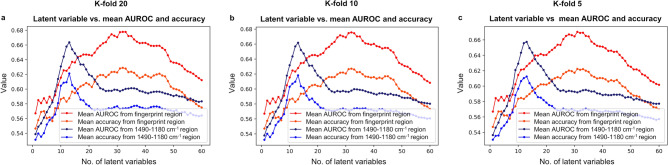
Mean AUROC and mean accuracy values against the latent variables in case of the fingerprint region (1800–900 cm^−1^) and region of 1490–1180 cm^−1^. Results from (**a**) k-fold 20, (**b**) k-fold 10 and (**c**) k-fold 5 have been presented. As seen from the figure, 32 latent variables yielded the best performance when fingerprint region was studied, and k-fold was 20 or 10, but for k-fold 5 the 31 latent variables were the best possible choice. In case of region of 1490–1180 cm^−1^, the 13 latent variables showed the best performance for k-folds 20, 10 and 5.

**Figure 5:**
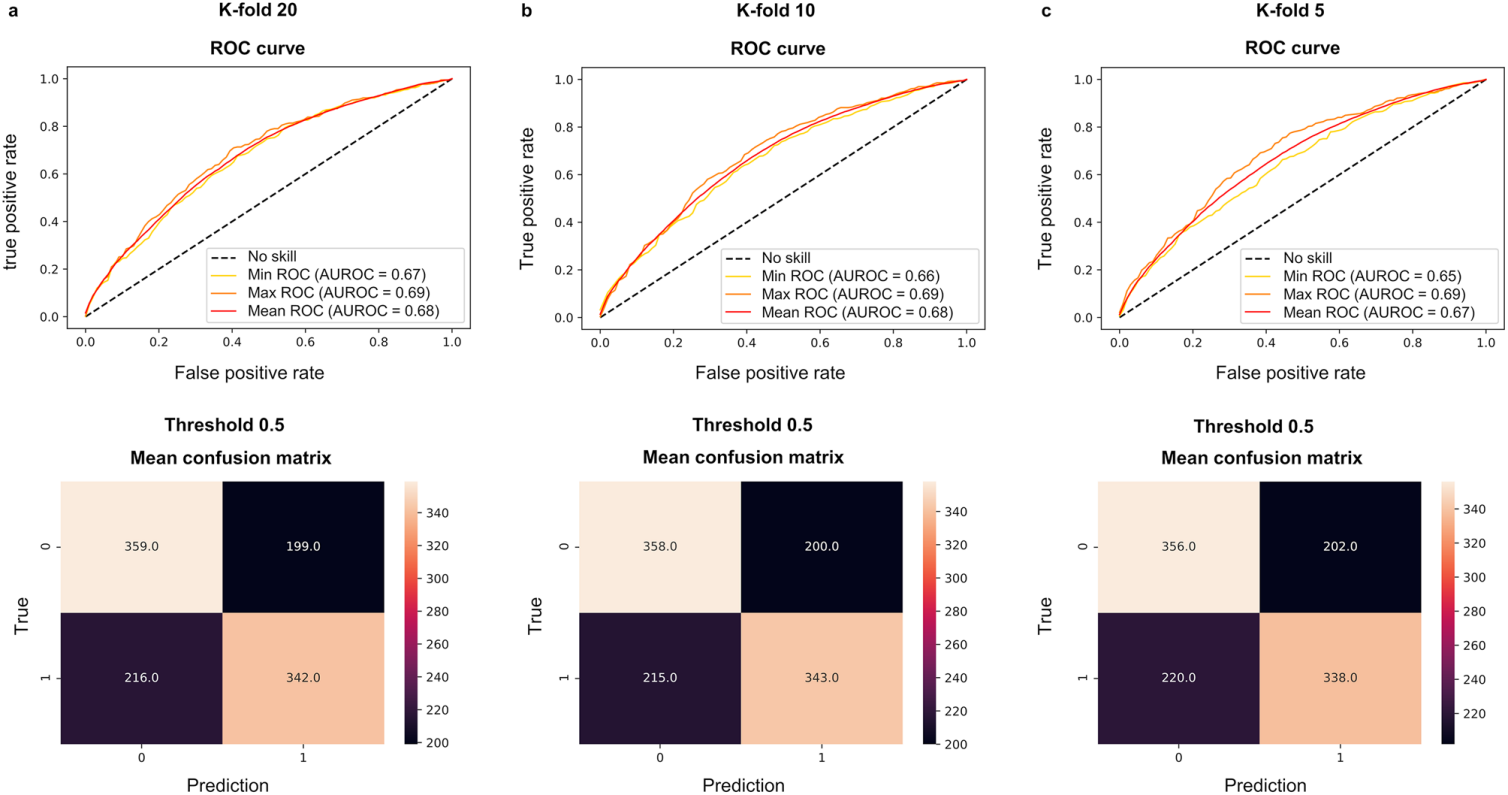
Averaged ROC curves and confusion matrices from the PLS-DA analysis. Results from (**a**) k-fold 20, (**b**) k-fold 10 and (**c**) k-fold 5 have been presented. The PLS-DA k-fold cross-validation predictive ability was repeated 100 times with k-folds 20, 10 and 5. Training was accomplished with ATR-FTIR data from the spectral region of 1800–900 cm^−1^.

There were 1116 nasopharyngeal swab samples from separate patients, of whom 53% (592 of 1116) of patients were female (Fig. 2). The patients were having ages between 0 and 94 years (mean ± standard deviation, 32 years ± 19 years). The dataset containing 1116 ATR-FTIR spectra from the 558 positive and 558 negative nasopharyngeal swab samples, dissolved into the viral transport medium, and inactivated by adding the viral lysis buffer liquid, was analyzed with the PLS-DA method, with PCR analysis as the gold standard method. In the spectral data, the averaged spectra (the mean spectrum from three repetitive measurements per one sample) from the fingerprint region (Fig. 3) fed for the PLS-DA model yielded the best results. When the best PLS-DA model performance was obtained, there were 32 latent variables set for the model in case of k-folds 20 and 10, but for k-fold 5 the 31 latent variables were optimal choice (Fig. 4). The mean AUROC values of 0.68 (min 0.67, max 0.69), 0.68 (min 0.66, max 0.69) and 0.67 (min 0.65, max 0.69) were achieved from the PLS-DA k-fold cross-validation trainings repeated 100 times for k-folds 20, 10 and 5, respectively (Table 1). Obtained P-value for each k-fold showed significant difference between the mean AUROC value achieved in our study, and the mean AUROC value obtained from the same study repeated with the randomly permuted sample labels (for each P-value, P < 0.001). Averaged ROC curves were also calculated (Fig. 5).

**Table 1:** Results from the PLS-DA analysis of spectrum region of 1800–900 cm^−1^.

K-Fold	Mean AUROC	Min AUROC	Max AUROC	95% CI AUROC
20	0.68	0.67	0.69	[0.68, 0.68]
10	0.68	0.66	0.69	[0.67, 0.68]
5	0.67	0.65	0.69	[0.67, 0.67]

K-Fold	Mean accuracy	Min accuracy	Max accuracy	95% CI accuracy
20	0.63	0.62	0.64	[0.63, 0.63]
10	0.63	0.61	0.65	[0.63, 0.63]
5	0.62	0.60	0.64	[0.62, 0.62]

K-Fold	Mean specifity	Min specifity	Max specifity	95% CI specifity
20	0.64	0.62	0.66	[0.64, 0.65]
10	0.64	0.62	0.67	[0.64, 0.65]
5	0.64	0.59	0.66	[0.63, 0.64]

K-Fold	Mean sensitivity	Min sensitivity	Max sensitivity	95% CI sensitivity
20	0.61	0.59	0.63	[0.61, 0.62]
10	0.61	0.59	0.64	[0.61, 0.62]
5	0.61	0.58	0.65	[0.61, 0.61]

K-Fold	Mean precision	Min precision	Max precision	95% CI precision
20	0.63	0.62	0.65	[0.63, 0.64]
10	0.63	0.62	0.65	[0.63, 0.63]
5	0.63	0.61	0.65	[0.62, 0.63]

Averaged results from the PLS-DA model k-fold cross-validation predictive ability repeated 100 times with k-fold values 20, 10 and 5. For each mean AUROC value the P-value was P < 0.001, and the P-values were calculated by using test 1 according to Ojala and Garriga^22^. The number of latent variables were set to 32 for k-folds 20 and 10, but for k-fold 5 the latent variables were set to 31, as it was yielding the best performance in predictive ability. There were 558 negative and 558 positive nasopharyngeal swab samples (dissolved into the viral transport medium and inactivated by adding viral lysis buffer liquid) measured with ATR-FTIR spectroscopy. Every sample was measured three times. The measured samples were preprocessed before PLS-DA, by applying spectrum truncation to the spectrum region of 1800–900 cm^−1^, vector normalization and spectrum averaging to create a one average spectrum for every measured nasopharyngeal swab sample.

When the mean accuracies, the mean specificities, the mean sensitivities, and the mean precisions were calculated, a global threshold of 0.5 was set to divide the PLS-DA analyzed samples into the positive and negative classes, also resulting the mean confusion matrixes shown in Fig. 5. The mean accuracies of 0.63, 0.63, 0.62, and the mean specificities of 0.64, 0.64 and 0.64 for k-folds 20, 10 and 5, respectively were achieved (Table 1). The mean sensitivities of 0.61, 0.61, 0.61, and the mean precisions of 0.63, 0.63 and 0.63 were obtained for k-folds 20, 10 and 5, respectively (Table 1).

The spectral region of 1490–1180 cm^−1^ (Fig. 3) from fingerprint region yielded results close to, or almost the same compared to the fingerprint region when the 13 latent variables were set for the PLS-DA model (Fig. 4). The achieved mean AUROC values from the PLS-DA training were 0.66 (min 0.66, max 0.67), 0.66 (min 0.65, max 0.67) and 0.66 (min 0.63, max 0.67) for k-folds 20, 10 and 5, respectively (Table 2). Obtained P-value for each k-fold was showing significant difference between the mean AUROC value from the study of the spectral region of 1490–1180 cm^−1^, and from the same study repeated with randomly permuted sample labels (for each P-value, P < 0.001).

**Table 2:** Results from the PLS-DA analysis of spectrum region of 1490–1180 cm^−1^.

K-Fold	Mean AUROC	Min AUROC	Max AUROC	95% CI AUROC
20	0.66	0.66	0.67	[0.66, 0.66]
10	0.66	0.65	0.67	[0.66, 0.66]
5	0.66	0.63	0.67	[0.66, 0.66]

K-Fold	Mean accuracy	Min accuracy	Max accuracy	95% CI accuracy
20	0.62	0.61	0.63	[0.62, 0.62]
10	0.62	0.60	0.64	[0.62, 0.62]
5	0.61	0.59	0.63	[0.61, 0.61]

K-Fold	Mean specifity	Min specifity	Max specifity	95% CI specifity
20	0.63	0.61	0.65	[0.63, 0.63]
10	0.63	0.61	0.65	[0.63, 0.63]
5	0.63	0.60	0.66	[0.63, 0.63]

K-Fold	Mean sensitivity	Min sensitivity	Max sensitivity	95% CI sensitivity
20	0.61	0.59	0.63	[0.61, 0.61]
10	0.60	0.58	0.63	[0.60, 0.61]
5	0.60	0.56	0.63	[0.59, 0.60]

K-Fold	Mean precision	Min precision	Max precision	95% CI precision
20	0.62	0.61	0.64	[0.62, 0.62]
10	0.62	0.61	0.64	[0.62, 0.62]
5	0.62	0.60	0.64	[0.61, 0.62]

Averaged results from the PLS-DA model k-fold cross-validation predictive ability repeated 100 times with k-fold values 20, 10 and 5. For each mean AUROC value the P-value was P < 0.001, and the P-values were calculated by using test 1 according to Ojala and Garriga^22^. The number of latent variables were set to 13, as it was yielding the best performance in predictive ability. There were 558 negative and 558 positive nasopharyngeal swab samples (dissolved into the viral transport medium and inactivated by adding viral lysis buffer liquid) measured with ATR-FTIR spectroscopy. Every sample was measured three times. The measured samples were preprocessed before PLS-DA, by applying spectrum truncation to the spectrum region of 1490–1180 cm^−1^, vector normalization and spectrum averaging to create a one average spectrum for every measured nasopharyngeal swab sample.

## Discussion

The aim of our study was to investigate whether the diagnostic performance of the ATR-FTIR spectroscopy coupled with PLS-DA is adequate to detect SARS-CoV-2 infection from the nasopharyngeal swab samples originally collected for PCR analysis. The AUROC values obtained in our study showed moderate performance (the AUROC values were between 0.66 and 0.68) when the fingerprint region, and the spectral region of 1490–1180 cm^−1^ were studied. Analyzed dataset contained ATR-FTIR spectra from the 558 negative and 558 positive samples from the separate patients within a real clinical setting in the city of Oulu, Finland.

When analyzing results from our study, it seems that the spectral region of 1490–1180 cm^−1^ is containing enough information to classify ATR-FTIR spectra, even though the whole fingerprint region (1800–900 cm^−1^) is often used in earlier studies. On the other hand, it was not possible to find out separate wavenumbers that could be used to classify spectra. Therefore, at least in this dataset, the meaningful information is spread out over certain spectral region rather than just focused on the separate wavenumbers (see Supplementary Fig. S1). Moreover, it is notable that the spectral region of 1490–1180 cm^−1^ was classified with the 13 latent variables, whereas the 31–32 latent variables were needed in case of the whole fingerprint region (1800–900 cm^−1^). Less latent variables mean simpler model that usually refers to better generalization ability. Consequently, in that sense the spectral region of 1490–1180 cm^−1^ could be more suitable choice for spectral analysis.

We observed that only simple spectral preprocessing was needed to prepare the mean spectra for classification, i.e., spectrum truncation to the fingerprint region or the region of 1490–1180 cm^−1^, vector normalization, and spectrum averaging. In practice, this means that our workflow to classify spectra is relatively easy to set up, and the retraining of PLS-DA model in case of more training data available could be also easily accomplished.

Our results with ATR-FTIP spectroscopy showed clearly weaker performance than the results of previous studies with similar methods focusing on SARS-Cov-2 detection. Zhang et al.^10^ studied serum with ATR-FTIR spectroscopy using a total of 115 samples (41 of them were confirmed to be COVID-19 positive and others were from healthy donors and patients with other infections or inflammatory diseases). They analyzed the measured dataset with the PLS-DA method and reported the AUROC value as high as 0.9561. Furthermore, coronavirus detection from the saliva (pharyngeal swabs) was conducted by collecting a total of 111 negative and 70 positive samples by combining genetic algorithm with linear discriminant analysis (GA-LDA). Results from that saliva study showed 90% accuracy, 95% sensitivity and 89% specificity^12^. Other saliva-based study was performed by asking donors to dribble into a container with added viral transport medium and by analyzing data with the PLS-DA method. From the collected samples, 29 were confirmed as positive and 28 as negative (in overall there were 171 transflection infrared spectra) yielding the sensitivity of 93%, and the specificity of 82%^13^. In a very recent study, ATR-FTIR spectroscopy coupled with partial least squares (PLS) and cosine k-nearest neighbours (KNN) analysis was applied to detect SARS-Cov-2 from nasopharyngeal swab^14^. There were samples from 243 patients (in total of 714 ATR-FTIR spectra), as where 40 + 111 patients were confirmed as COVID-19 positive. Samples were inserted into the viral transport medium 1 (the liquid 1) or the viral transport medium 2 (the liquid 2). For the liquid 1, the sensitivity was reported to be 84%, the specificity 66% and the accuracy 76.9%. In the case of liquid 2, the sensitivity was 87%, the specificity 64% and the accuracy 78.4%.

There can be various reasons for the overall weaker performance of ATR-FTIR spectroscopy observed in our study. First and foremost, as the nasopharyngeal swab sample was dissolved into a relatively large amount of solvent, the low concentration of viral particles in the viral transport medium (and the viral lysis buffer) may explain the lower diagnostic performance. In addition, it should be taken account that the swab samples were collected also from the people without symptoms but been exposed to COVID-19. It is possible that the performance would be different if the swab samples had been collected from the hospitalized patients of whom swab samples typically contains higher viral loads and the control samples would have been collected from the healthy volunteers.

As a second reason, even though the sample was vortexed before the ATR-FTIR measurements, the sample solution may still have distributed unevenly onto the ATR crystal. This assumption about the uneven viral material distribution may be reinforced by the observation from preliminary test that the averaged spectra (from three repetitive measurements) provided better diagnostic performance when compared to training and validation with each separate spectrum. Consequently, it is possible that the used viral transport medium (and the viral lysis buffer) affects samples causing them to be unevenly distributed, which induces unwanted variation in the ATR-FTIR measurements. In optimal situation, liquid sample drops should be as homogenous as possible to obtain the most accurate results.

It may also well be that the physical sensitivity of ATR-FTIR spectroscopy with these low concentration levels is inadequate to reach the high levels of diagnostic performance. This speculation is also supported by the recent study where excellent diagnostic performance was reported (the accuracy of 90%) when ATR-FTIR spectroscopic measurements were conducted directly from the pharyngeal swab samples, i.e., without dissolving the sample to the viral transport media^12^. If that is truly the case, ATR-FTIR spectroscopy could not be recommended as the SARS-CoV-2 screening tool from the swab samples dissolved into the viral transport medium. Instead, the measurement should be conducted directly from the swab stick, or at least without any added chemicals.

Finally, storing and freezing of samples could be one limiting factor, as it is possible for RNA molecules to lose their structural integrity and degrade after long storage period^24^. This could alter the size of native and viral RNA fragments and the composition of covalent bonds in samples taken at different times, resulting in heterogeneity of samples. Since difference in nucleic acid content, specific to the virus, is a major factor for an accurate assessment of ATR-FTIR, effects of storing samples should be taken account. Obviously, it would have been the best to conduct all the ATR-FTIR spectroscopy measurements on the same day as the PCR analysis without freezing the samples in between. Unfortunately, it was not logistically possible in this study.

In this study, we used a constant global threshold (0.5) in PLS-DA model to separate between the negative and positive classes. Obviously, if selecting different threshold, the mean accuracies, the mean specificities, the mean sensitivities, and the mean precisions would be different. If portable and fast SARS-CoV-2 detecting test would be developed in the future, the practical choice could be to maximize the specificity, i.e., the ability to detect persons without the disease as effectively as possible. Then persons with negative test result could be diagnosed to be healthy with high confidence. And if positive result would be achieved, one possibility could be then to send a person for PCR test. That would be the case especially if the sensitivity, which describes the ability to detect persons with disease, would be slightly lower.

The indisputable strength of our study is the high number of investigated samples and the balance between COVID-19 positive and negative samples when compared to other studies (our study included a total of 1116 separate nasopharyngeal swab samples), including the studies by Nogueira et al. with a total of 243 nasopharyngeal swab samples in the viral transport medium and Barauna et al. with a total of 181 undiluted pharyngeal swab samples^12,14^. It is well known that the performance values can change, sometimes even drastically, after the preliminary analysis with smaller sample set compared with the eventually larger dataset. Actually, even during our sample collection, we observed better diagnostic performance values when we had collected only less than 200 samples in total (the AUROC values around 0.75–0.78). However, when the sample size increased, the performance values started to stabilize to the current level (the AUROC values between 0.66 and 0.68). Consequently, more studies with the representative sample sizes (preferably several thousands) are needed to truly validate the ATR-FTIR spectroscopy method for clinical diagnostics of SARS-CoV-2 infection. Also, different ways to create stronger IR-signal to better separate between samples in different classes should be studied carefully. Obviously, best choice would be to study samples with higher viral concentrations. In addition, multi-reflection ATR could be one way to amplify the magnitude of absorbance bands in measured spectrums.

## Conclusions

As our study was containing relatively large amount of nasopharyngeal swab samples and the amount of COVID-19 positive and negative samples was balanced (${\text{n}}_{\text{positive}}$ = 558 and ${\text{n}}_{\text{negative}}$ = 558), it could be expected that our study can offer more realistic estimate of the performance of the ATR-FTIR measurement coupled with machine learning -based analysis than the earlier studies. The conclusion is that the ATR-FTIR measurement with machine learning -based analysis can detect the SARS-CoV-2 infection with moderate performance when taken from the samples intended for the PCR-measurement and inactivated by using the viral lysis buffer. The moderate performance might be related to the low concentration of the viral particles in the transport medium and the viral lysis buffer. Still more studies with more samples will be needed to better justify the diagnostic performance of the discussed SARS-CoV-2 detecting method. Particularly, studying another type of virus would step up proofing of the usefulness of the ATR-FTIR method.

## Supplementary Information

Supplementary Figure S1.

## Data Availability

The datasets generated during and/or analysed during the current study are available from the corresponding author on reasonable request.
