# Supplementary material for: Diagnostic performance of attenuated total reflection Fourier-transform infrared spectroscopy for detecting COVID-19 from routine nasopharyngeal swab samples
Source: Sci Rep. 2022 Nov 27;12:20358. doi: 10.1038/s41598-022-24751-z (PMC9701801; doi:10.1038/s41598-022-24751-z)
Supplement: Supplementary file 1 — Supplementary Figure S1. [file 41598_2022_24751_MOESM1_ESM.pdf]

<u>**Supplementary Information**</u>

Diagnostic performance of attenuated total reflection Fourier-transform infrared spectroscopy for detecting COVID-19 from routine nasopharyngeal swab samples

Helinä Heino[1], Lassi Rieppo[1], Tuija Männistö[2], Mikko J. Sillanpää[3],

Vesa Mäntynen[2], Simo Saarakkala[1,4]

[1]Research Unit of Medical Imaging, Physics and Technology, University of Oulu, Oulu, Finland

[2]Northern Finland Laboratory Centre NordLab, NordLab Oulu, Oulu, Finland

[3]Research Unit of Mathematical Sciences, University of Oulu, Oulu, Finland

[4]Department of Diagnostic Radiology, Oulu University Hospital, Oulu, Finland

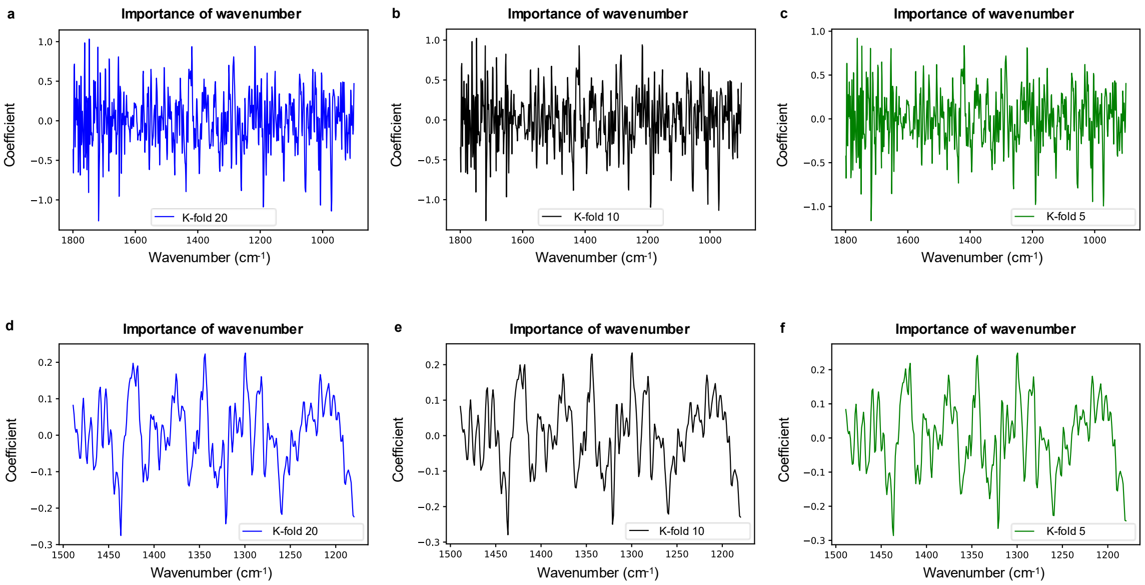

Supplementary Figure S1. Importance of wavenumber. Coefficients from PLS-DA model against wavenumbers have been presented to visualize importances of wavenumbers in classification. Visualized coefficients are averaged coefficients obtained from different k-fold trainings. Results from **a)** k-fold 20, **b)** k-fold 10 and **c)** k-fold 5 trainings from fingerprint region (1800-900 cm^{-1}) have been presented. Also, results from **d)** k-fold 20, **e)** k-fold 10 and **f)** k-fold 5 trainings from region of 1490-1180 cm^{-1} have been included. As seen from the figure, there were multiple important wavenumbers when fingerprint region and region of 1490-1180 cm^{-1} were analyzed.
